# Hexane Fractions of *Bupleurum falcatum L.* Stimulates Glucagon-Like Peptide-1 Secretion through G_**βγ**_-Mediated Pathway

**DOI:** 10.1155/2014/982165

**Published:** 2014-02-13

**Authors:** Min-Hee Shin, Eun-Kyeong Choi, Ki-Suk Kim, Kang-Hoon Kim, Young Pyo Jang, Kwang Seok Ahn, Won-Seok Chung, Nam Hyun Cha, Hyeung-Jin Jang

**Affiliations:** ^1^College of Korean Medicine, Institute of Korean Medicine, Kyung Hee University, 1 Heogi-dong, Dongdaemun-gu, Seoul 130-701, Republic of Korea; ^2^College of Pharmacy, Kyung Hee University, Heogi-dong, Dongdaemun-gu, Seoul 130-701, Republic of Korea; ^3^Nursing Department, College of Health Science, Kangwon University, Hyoja 2-dong, Chuncheon-si 200-701, Republic of Korea

## Abstract

*Bupleurum falcatum L.* has been used traditionally as a medicinal herb in Korean medicine. The hexane fraction of BF (HFBF), which was profiled with Direct Analysis in Real Time-Mass Spectrometry (DART-MS), activates the secretion of glucagon-like peptide-1 (GLP-1) in NCI-H716 cells significantly. We performed a microarray analysis and GLP-1 ELISA assay, as well as calcium imaging experiments with inhibitors, to investigate the mechanism of action of the HFBF. Through the microarray analysis, it was found that the *ITPR2* gene that encodes the inositol 1,4,5-trisphosphate (IP_3_) receptor is up-regulated and the HFBF induces cell depolarization by inhibiting the voltage-gated channel expression in NCI-H716 cells. In addition, we found that the intracellular calcium in NCI-H716 cells, with Gallein, U73122, and 2APB as inhibitors, was decreased. These results suggest that the HFBF activates the GLP-1 secretion through the G_*βγ*_ pathways in the enteroendocrine L cells after treatment with the HFBF.

## 1. Introduction

*Bupleurum falcatum L.* (BF) has been used traditionally as a medical herb [1]. It is used for the preparation of herbal remedies and also used as an ingredient in herbal tea and traditional fermented beverages. BF is also known for its therapeutic effects in the treatment of diabetes [1]. Despite its outstanding effects during clinical trials on diabetes mellitus, its mode of action has not been examined. To study the effects of a herbal sample, BF was extracted and fractionated as described in [2, 3]. The hexane fractions of *Bupleurum falcatum L.* (HFBF) samples were used to treat enteroendocrine NCI-H716 cells and subsequently a GLP-1 ELISA was performed. The microarray was also examined using isolated RNA from HFBF treated NCI-H716 cells.

The NCI-H716 cell line, the human intestinal cell, is widely used to study glucagon-like peptide-1 (GLP-1) secretion [4, 5]. GLP-1 is an incretin hormone that is released by enteroendocrine L cells in the gastrointestinal tract (GI) and has received considerable interest because of its ability to amplify insulin secretion in pancreatic *β*-cells [6]. GLP-1 is secreted within minutes of nutrient ingestion and boosts the disposal of ingested nutrients.

Seven-transmembrane receptor guanine nucleotide-binding protein (G protein) coupled receptor (GPCR), also known as a G protein-linked receptor, is composed of G_*α*_, G_*β*_, and G_*γ*_. Both G_*α*_ and G_*βγ*_ are related to the GPCR signaling pathway. The G_*α*_ subunit is related to the cyclic AMP (cAMP), protein kinase A (PKA), and phosphodiesterases (PDEs). In addition, the G_*βγ*_ subunit activates the phospholipase C *β* (PLC *β*) that separates the phospholipid phosphatidylinositol 4,5-bisphosphate (PIP_2_) into diacyl glycerol (DAG) and inositol 1,4,5-trisphosphate (IP_3_). IP_3_ then disperses through the cytosol to bind to IP_3_ receptors, particularly in the calcium channels in the smooth endoplasmic reticulum (ER). This leads to the increase of Ca^2+^ concentration in the cytosol and causes a cascade of intracellular changes and activities. Also, Ca^2+^ and DAG together activate the protein kinase C (PKC). Hormone secretion such as GLP-1 is highly related to the intracellular Ca^2+^ signaling, which is generally related to the two processes. One is the Ca^2+^ influx due to the depolarization and transmembrane potassium channels. The other is Ca^2+^ release from the intracellular calcium storage area including the endoplasmic reticulum [7].

The G protein-gated ion channels are specific ion channels located in the plasma membrane that are activated by a family of associated proteins. These ion channels allow for the selective movement of certain ions across the plasma membrane in cells. Incretin hormones modulate voltage-gated potassium (Kv) channels [2, 3]. Incretin hormones like GLP-1 also activate the closure of ATP-sensitive potassium channels resulting in membrane depolarization and the activation of voltage-dependent Ca^2+^ channels resulting in the increase in intracellular Ca^2+^. GLP-1 stimulates insulin secretion through the modulation of the ATP-dependent potassium channel, increasing the Ca^2+^ influx and releasing Ca^2+^ from intracellular stores [8].

In this study, we demonstrate that HFBF induces GLP-1 secretion via the GPCR signaling pathway, especially through the G_*βγ*_-mediated pathway. This study provides the important information of the HFBF effects to promote GLP-1 secretion and the possibility that the medical herb can be used as a therapeutic agent of diabetes mellitus.

## 2. Materials and Methods

### 2.1. Preparation of *Bupleurum falcatum L*. Extracts

The herbal extracts and fractions were purchased from Kyung-Hee Oriental Herbal Medicine Research Center. The BF samples were prepared as described in [9, 10]. Briefly, the rind parts of BF were extracted with distilled water (DW) and filtration, evaporation, and freeze drying were performed in order. The extracts were successively partitioned with organic solvents of different polarities to yield the *n*-hexane (HX) fractions.

### 2.2. Culture of NCI-H716 Cells

Enteroendocrine NCI-H716 cells were purchased from the Korean Cell Line Bank (KCLB, South Korea) and cultured in RPMI 1640 (Welgene, South Korea) with 10% FBS (Welgene, South Korea), penicillin, and streptomycin. To perform the endocrine differentiation, NCI-H716 cells were incubated in the Matrigel- (BD Bioscience, USA) coated 12-well plates,  1 × 10^6^ cells per well with high glucose DMEM (Welgene, South Korea) with 10% FBS, penicillin, and streptomycin. 48 hours later, the media were removed and the plates were washed with PBS, and then the cells were starved with low glucose DMEM (Welgene, South Korea). After 12 hours, the media were removed and the plates were washed again with PBS. 900 **μ**L of 1 mM calcium chloride PBS was added to each well and 100 **μ**L of each sample was (10x) treated for 1 hour in a CO_2_ incubator.

### 2.3. DART-MS Analysis of the Hexane Fraction of *Bupleurum falcatum L*.

A DART ion source (Ion Sense, Saugus, USA) combined with JMS-T100TD was used in the positive ion mode. Analysis conditions were set as needle voltage 3200 V, electrode 1, 2 voltage 100 V, helium gas flow 3 L/min, temperature 250°C, the first orifice lens 15 V, and ring lens voltage 5 V. Analyzed molecular weight peaks were normalized with polyethylene glycol 600 (PEG 600) [10, 11].

### 2.4. Cell Viability Assay

The 1 × 10^4^ NCI-H716 cells were seeded in 96-well plate after matrigel coating. After two days of differentiation and starvation overnight, BFHF were treated with three concentrations: 10 *μ*g/mL, 100 *μ*g/mL, and 1000 *μ*g/mL. MTT(3-(4,5-dimethythiazol-2-yl)-2,5-Diphenyl tetrazolium bromide) was obtained from Invitrogen (M6494, USA). Before treatment, it was dissolved as a 1 mg/mL stock and treated with cells. After 2 hours, the supernatants were sucked and 100 *μ*L DMSO was added. After shaking for 20 minutes, read plate with wavelength of 570 nm.

### 2.5. GLP-1 Secretion Study

Three days before the experiments, the cells were seeded at 1 × 10^6^ cells/well into 12-well culture plates precoated with Matrigel (BD Biosciences, USA). One day before the experiments, serum starvation was carried out with a DMEM low glucose medium for 20 hours. On the day of the experiments, the supernatants were replaced by PBS containing 1 mM CaCl_2_. Cells were incubated for 1 hour at 37°C with the different fractions of BF, and the GLP-1 was measured using a GLP-1 ELISA Kit (Millipore, USA). The data was normalized with total protein concentrations corresponding to each sample well.

### 2.6. Calcium Imaging

Differentiated NCI-H716 cells grown on cover glass were incubated overnight. Cytosolic free calcium [Ca^2+^] was measured using fura-2 fluorescence dye. NCI-H716 cells grown on a matrigel-coated cover-slide bottom dish were washed three times with PBS and incubated in the dark for 30 min at room temperature with fura-2AM (final concentration 1 *μ*M) in PBS. The cells were again washed with PBS three times and analyzed by being illuminated with alternating light of 340 and 380 nm from a rotating filter wheel.

### 2.7. RNA Isolation

Experiments were carried out in similar GLP-1 secretion study processes and we used 100 *μ*g/mL of the HFBF. Three days before the experiments, the cells were seeded at 1 × 10^7^ cells/well into 6-well culture plates precoated with Matrigel (BD Biosciences, USA). Other procedures were performed in the same way as described in the GLP-1 assay. Total RNA was extracted by using Hybrid-^RTM^ (GeneAll Biotechnology, Korea) according to the manufacturer's instructions. The microarray of each total RNA sample (200 ng) was labeled and amplified using a Low Input Quick Amp Labeling Kit (Agilent Technologies, USA) [10], and the Cy3-labeled aRNAs were resuspended in 50 **μ**L of hybridization solution (Agilent Technologies, USA). After the aRNAs were placed on an Agilent SurePrint G3 Human GE 8 × 60 K Array (Agilent Technologies, USA) and covered by a Gasket 8-plex slide (Agilent Technologies, USA), the slides were hybridized for 17 hours in a 65°C oven. The hybridized slides were washed in 2 × saline-sodium citrate (SSC), 0.1% sodium dodecyl sulfate (SDS) for 2 min, 1 × SSC for 3 min, and then 0.2 × SSC for 2 min at room temperature. The slides were centrifuged at 3000 rpm for 20 sec to dry.

### 2.8. Microarray of NCI-H716 Cells

Each total RNA sample (200 ng) was labeled and amplified using a Low Input Quick Amp Labeling Kit (Agilent Technologies, CA). The Cy3-labeled aRNAs were resuspended in 50 *μ*L of hybridization solution (Agilent Technologies, CA). After the labeled aRNAs were placed on an Agilent SurePrint G3 Human GE 8 × 60 K array (Agilent Technologies, CA) and covered by a Gasket 8-plex slide (Agilent technologies, CA), the slides were hybridized for 17 hours in a 65°C oven. The hybridized slides were washed in 2 × saline-sodium citrate (SSC), 0.1% sodium dodecyl sulfate (SDS) for 2 min, 1 × SSC for 3 min, and then 0.2 × SSC for 2 min at room temperature. The slides were centrifuged at 3000 rpm for 20 sec to dry.

### 2.9. Data Analysis

The arrays were analyzed through the use of an Agilent scanner and the associated software and gene expression levels were estimated with the Feature Extraction v10.7.3.1 (Agilent Technologies, USA). Relative signal intensities for each gene were generated using the Robust Multiarray Average Algorithm. The data were processed according to a quantile normalization method using the GeneSpring GX 11.5.1 (Agilent Technologies, USA). This normalization method used for making the distribution of intensities for each array in the set of arrays was the same. The normalization and log transformed intensity values were analyzed using the GeneSpring GX 11.5.1 (Agilent Technologies, USA). Fold change filters included the requirement that the genes are present in at least 2-fold of the controls for upregulated genes and less than 0.5-fold of the controls for downregulated genes.

### 2.10. Oral Glucose Tolerance Test

The *db/db* mouse was purchased from DBL (Korea). The *db/db* mouse (*n* = 5) was fasted for 16 hours and then orally administered at the volume of 5 g/kg glucose solution [12]. HFBF was treated at the volume of 100 mg/kg before treatment of the glucose. Blood samples, were obtained through the tail vein for 6 time points: 0 (before the HFBF administration), 10, 20, 40, 90, and 120 min after the glucose injection for the determination of blood glucose levels. Blood samples were obtained before gavage (time 0) and 10, 20, 40, 9, and 120 min after gavage for determination of blood glucose by ACCU-CHEK Performa System (Roche, South San Francisco, CA) [13].

### 2.11. Statistical Analysis

Each ELISA and calcium imaging set of data represents at least two separate experiments and each experiment was performed in triplicate. The significance of the data was analyzed with Prism 5 software with one-way ANOVA and Bonferroni tests to compare each set of data. Bars show the SEMs of the means of the three assays.

## 3. Results

### 3.1. DART-MS Analysis of Hexane Fraction of *Bupleurum falcatum L*.

**Figure 1:**
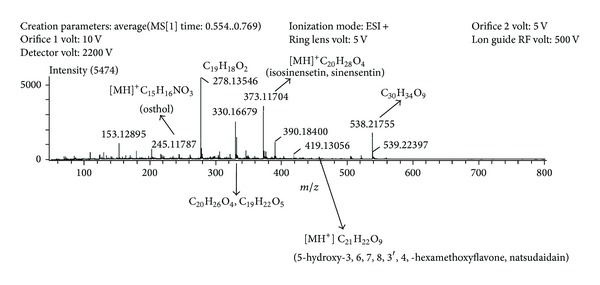
DART-MS profiling of HFBF.

DART-MS was performed to profile the HFBF (Figure 1). Among the several prominent peaks, isosinensetin (sinensetin) was identified as the second highest peak at m/z 194 ([MH]^+^ = 373.11704). DART-MS results show isosinensetin is a candidate for the active compound of the HFBF which contributes to the GLP-1 secretion effect. A study using isosinensetin may increase the potential of the HFBF as a therapeutic agent for type 2 diabetes mellitus.

### 3.2. HFBF Activates Secretion of GLP-1 in NCI-H716 Cells without Cytotoxicity

**Figure 2:**
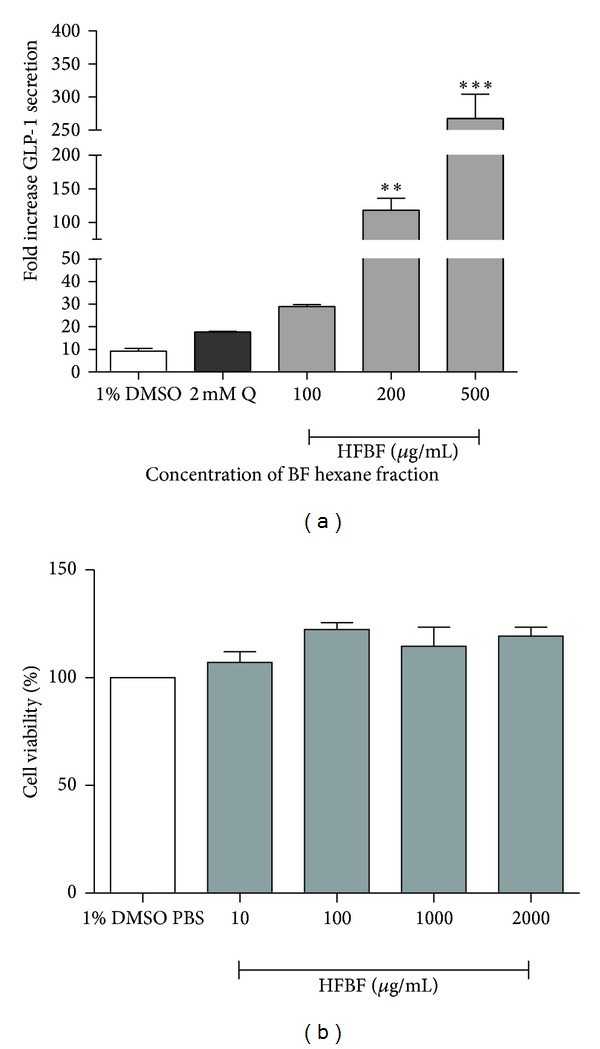
HFBF induced the secretion of GLP-1 in enteroendocrine cells without cell damage. BF extracted was fractionated with *n*-hexane (HX). (a) HFBF was treated with 100, 200, and 500 *μ*g/mL each to the differentiated NCI-H716 cells. GLP-1 secretion was compared with the negative control and positive control. 1% DMSO used as negative control and 2 mM Quinine (Q) used as positive control. Experiments were conducted in triplicate and normalized with protein contents. (b) The effect of HFBF on the cell viability was measured by MTT test. All concentrations of HFBF had no toxicity to the NCI-H716 cell. Statistical significance was determined by a one-way ANOVA and the values are means ± SEM; *P < 0.05, **P < 0.001, ***P < 0.0005.

To confirm the GLP-1 secretion effect due to the HFBF treatment, a GLP-1 assay was conducted. The extracts were diluted with distilled water and were successively partitioned with organic solvents of different polarities as described in the Materials and Methods section. For GLP-1, an ELISA was conducted using a BF fraction including butanol, ethyl acetate, dichloromethane, and hexane. The HFBF activated GLP-1 secretion at higher levels compared to the other extracts (data not shown). As shown in Figure 2(a), GLP-1 secretion was significantly increased in a dose dependent manner compared to the control; DMSO was used as a negative control and 2 mM Quinine was used as a positive control. 1% DMSO was used as a negative control because the entire compound dissolved in it. The HFBF increased GLP-1 secretion by almost 25-fold compared to the negative control.  Figure 2(b) shows HFBF has no cytotoxicity to the NCI-H716 cells.

### 3.3. Hexane Fraction of *Bupleurum falcatum L*. Induces GLP-1 Secretion through GPCR Pathways

**Figure 3:**
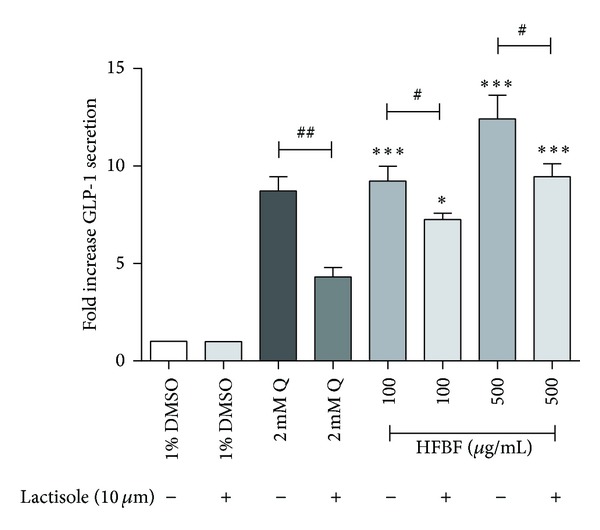
Inhibition study of GLP-1 secretion using lactisole. To test the inhibitory effect of the lactisole, lactisole was treated to the NCI-H716 cells. The BF hexane fraction (HFBF) was treated with 100, 200, and 500 *μ*g/mL each to the differentiated NCI-H716 cells. GLP-1 secretion was compared with the negative control and positive control. 1% DMSO used as negative control and 2 mM Quinine (Q) used as positive control. Statistical significance was determined by a one-way ANOVA and the values are means ± SEM; *P < 0.05 and ***P < 0.0001 versus 1% DMSO. ^#^ *P* < 0.05 and ^##^ *P* < 0.001 lactisole and HFBF treated group versus only HFBF treated group.

Microarray data demonstrated the HFBF activates GLP-1 secretion through GPCR signaling pathways in NCI-H716 cells. GPCR stimulates G protein that is composed of *α*, *β*, and *γ* subunits. G_*α*_ and G_*βγ*_ act independently through different signaling pathways. As shown in Table 1, the *ADCY1* gene that encodes AC is downregulated and *PDE*-related genes including *PDE8B*, *PDE8A*, *PDE7B*, *PDE4D*, *PDE4D*, *PDE4D*, *PDE4C*, *PDE4B*, *PDE4A*, and *PDE2A* are also downregulated. The *ITPR2* that encodes the inositol 1,4,5-trisphosphate receptor is upregulated (Table 1). To verify the activated pathway of the HFBF, an inhibition study was conducted. Lactisole, which is known as an inhibitor of sweet and umami taste receptors, was also used as an inhibitor of the G_*α*_ pathway [14]. There is no significant result in the inhibition study of GLP-1 secretion using lactisole (Figure 3). The HFBF seems to activate GLP-1 secretion through the G_*βγ*_-mediated pathway particularly with the activation of PLC. The upregulation of the *ITPR2* gene and downregulation of PDE and AC demonstrate that the HFBF stimulates GLP-1 secretion through the G_*βγ*_-mediated pathway [8, 15].

### 3.4. HFBF Induced Downregulation of Potassium Voltage-Gated Channels

The cell creates electrical signal through several types of ion channels such as potassium voltage-gated channels that are important to create membrane depolarization [16, 17]. *KCNC3, KCNA6, KCNQ2, KCNE1, KCNN1*, and *KCTD19* genes encode potassium voltage-gated channels and are downregulated by the HFBF (Table 2). The HFBF seems to induce GLP-1 secretion by changing membrane potential via the potassium voltage-gated channels. HFBF has effects on making cell depolarization and inhibiting the expression of potassium voltage-gated channels by the downregulation of *KCNC3, KCNA6, KCNQ2, KCNE1, KCNN1*,and *KCTD19* genes. This change of membrane potential may cause the secretion of hormones in enteroendocrine cells [18].

### 3.5. HFBF Induced GLP-1 Secretion through *G*_*βγ*_ Pathway

Lactisole is used as an inhibitor of sweet taste receptors that are known as a G_*α*_ pathway [19]. We treated lactisole to the NCI-H716 cell to verify the signaling pathway of the HFBF. As shown in Figure 3, lactisole showed no effect on blocking GLP-1 secretion during the treatment of the HFBF. This means the HFBF activates GLP-1 secretion but not through the G_*α*_ pathway. Thus, we performed calcium imaging experiments using Gallein, 2APB, and U73122 as other G_*βγ*_ signaling pathway inhibitors [19, 20].

**Figure 4:**
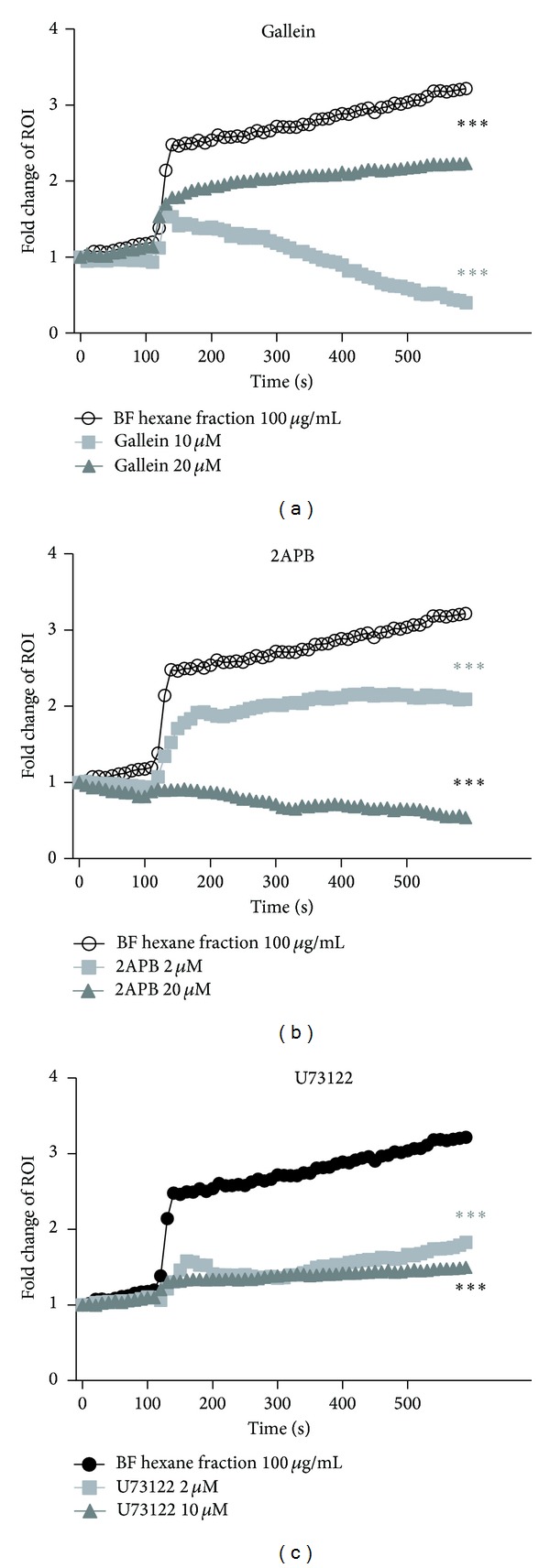
HFBF stimulates GLP-1 secretion through G_*βγ*_ pathway. Treatment of BF hexane fraction at 100 seconds into the medium increased the concentration of Ca^2+^ in cytosol. With BF hexane fraction, inhibitors were treated. (a) Gallein which is an inhibitor of G_*βγ*_ subunit-dependent signaling pathway reduced GLP-1 secretion dose dependently. (b) 2APB inhibits the IP_3_-induced Ca^2+^ release. 2APB also reduced GLP-1 secretion dose dependently. (c) U73122 is an inhibitor of PLC-dependent processes. With this inhibitor, concentration of Ca^2+^ showed less change despite treatment of BF hexane fraction. Data are means of intensity of the NCI-H716 cells (*n* = 10). ***P < 0.0001 versus BF hexane fraction 100 *μ*g/mL.

Intracellular Ca^2+^ is closely related to the GPCR signaling pathway [21]. Intracellular Ca^2+^ ion is produced naturally in the cell after the activation of GPCR. To confirm the signaling pathway of GLP-1 secretion, a G_*βγ*_ pathway inhibitor was treated. As shown in Figure 4(a), Gallein which is an inhibitor of the G_*βγ*_ subunit was treated to the NCI-H716 cell. Compared to the cells treated with only the HFBF, the Gallein-treated cells showed decreased intracellular calcium concentration. U73122, which is an inhibitor of PLC*β*2 activity, and 2APB, which is an inhibitor of both IP_3_ receptors and TRP channel treated cells, showed decreased intracellular calcium release in a dose dependent manner (Figures 4(b) and 4(c)). All of these results indicate the effect the HFBF has on GLP-1 secretion via the G protein *βγ* pathway.

### 3.6. Hexane Fraction of *Bupleurum falcatum L*. Decreases Blood Glucose Level in *db/db* Mouse

**Figure 5:**
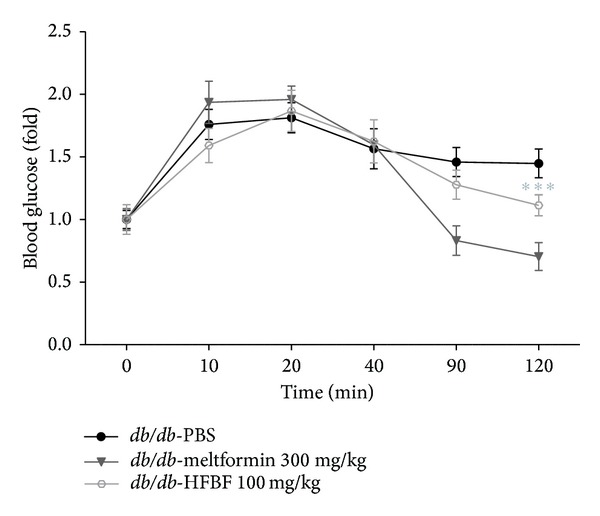
Oral glucose tolerance test of HFBF. To test regulatory effect of HFBF, HFBF treated orally to the *db/db* mouse. Metformin was used as a positive control. To compare with PBS-treated control group, 300 mg/kg of Metformin and 100 mg/kg of HFBF were orally treated just before the glucose administration. Mouse blood sample was collected from tail vein at 0 (before the HFBF treatment) and after 10, 20, 40, 90, and 120 minutes. Statistical significance was determined by student's *t*-test and the values are means ± SEM; ***P < 0.0001.

As shown in Figure 5, HFBF regulated the blood glucose level of *db/db* mouse. *db/db* mouse is a model of diabetes because it has no leptin receptor activity. It is point mutation in the gene for the leptin receptor. After glucose administration, blood glucose level increased time dependently before 20 min. However, HFBF administration group significantly shows the decreased glucose level compared to PBS-control group.

## 4. Discussion

This investigation suggests that HFBF stimulates GLP-1 secretion through the G_*βγ*_-mediated pathway. Glucagon-like peptide-1 (GLP-1) is an incretin hormone which regulates insulin secretion, appetite, and gut motility [4]. Lately, new drugs that help to release GLP-1 secretion or block the degradation of GLP-1 are issued because GLP-1 regulates the secretion of insulin in pancreatic *β*-cells. The major source of GLP-1 is the intestinal L cell that secretes GLP-1 as a gut hormone. In this investigation, HFBF shows the antidiabetic effect in cellular level by secreting GLP-1 secretion. HFBF was treated to NCI-H716 cells with three concentrations: 100, 200, and 500 *μ*g/mL. GLP-1 secretion was increased dose dependently. All concentrations of HFBF increased GLP-1 secretion and had significance. We supposed that the HFBF increases the GLP-1 secretion through sweet taste or bitter taste receptor signal pathway in enteroendocrine NCI-H716 cells. To verify this hypothesis. Lactisole was used as an inhibitor of sweet taste receptor, exactly, human T1R3. Sweet taste receptor is the heterodimer consisting of T1R2 and T1R3. After treatment of HFBF with lactisole, there is no significant effect comparing to the only HFBF treatment (Figure 3). G protein-coupled receptor is also composed of G_*βγ*_ subunit that is related to phospholipase C *β*2 (PLC *β*2), diacyl glycerol (DAG), and inositol 1, 4, 5-triphosphate (IP_3_) and increases intracellular calcium. Gallein, 2APB, and U73122 are inhibitors of G_*βγ*_ pathways [19, 20]. They inhibit G_*βγ*_ subunit, PLC *β*2, and IP_3_ receptor. The activated G_*βγ*_ activates PLC *β*2 and then divides PIP_2_ into DAG and IP_3_. DAG regulates protein kinase C (PKC) and triggers phosphorylation of PKC. IP_3_ is recruited to IP_3_ receptor in ER membrane and, as a result, releases calcium flow into cytoplasm. In Figure 4, Gallein decreased intracellular calcium concentration. U73122 and 2APB also showed decreased intracellular calcium release in a dose dependent manner. To verify the signaling pathway of GLP-1 secretion through the HFBF treatment, we performed microarray analyses. Microarray data show the HFBF activated GLP-1 secretion through the GPCR signaling pathway. HFBF and then following membrane depolarization. The data from the inhibition studies showed the HFBF activates the GPCR signaling pathway, especially the G_*βγ*_-mediated pathway, for the release of GLP-1. GLP-1 is one of target molecules for the treatment of Type 2 diabetes mellitus because it activates the GLP-1 receptor on the cell and then mediates insulin secretion. HFBF also showed regulatory effect of blood glucose level in *db/db* mouse OGTT experiments. This investigation suggests that HFBF stimulates GLP-1 secretion through G_*βγ*_-mediated signaling pathway. The overall conclusion from the results is that the HFBF could be used as a possible medication for Type 2 diabetes mellitus patients via activation of G_*βγ*_-mediated pathway.

## Figures and Tables

**Table 1:** List of genes related to the GPCR signaling pathway which is regulated after HFBF treatment.

Probe name	Description	Gene symbol	*P* value	Fold change
Inositol 1,4,5-trisphosphate				
A_33_P3298128	Inositol 1,4,5-trisphosphate receptor, type 2 (ITPR2), mRNA [NM_002223]	*ITPR2 *	0.004	**1.66 **

Adenylate cyclase				
A_23_P126313	Adenylate cyclase 10 (soluble) (ADCY10), transcript variant 1, mRNA [NM_018417]	*ADCY1 *	0.262	−**0.30 **

Phosphodiesterase				
A_24_P197537	Phosphodiesterase 8B (PDE8B), transcript variant 1, mRNA [NM_003719]	*PDE8B *	0.7777	**−0.04**
A_33_P3244951	Phosphodiesterase 8A (PDE8A), transcript variant 1, mRNA [NM_002605]	*PDE8A *	0.397	**−0.14**
A_33_P3301940	Phosphodiesterase 7B (PDE7B), mRNA [NM_018945]	*PDE7B *	0.238	**−0.22**
A_33_P3240552	Phosphodiesterase 4D, cAMP-specific [Source: HGNC Symbol; Acc: 8783] [ENST00000509355]	*PDE4D *	0.474	**−0.08**
A_33_P3389649	Phosphodiesterase 4D, cAMP-specific (PDE4D), transcript variant 4, mRNA [NM_001197218]	*PDE4D *	0.167	**−0.23**
A_33_P3389653	Phosphodiesterase 4D, cAMP-specific (PDE4D), transcript variant 3, mRNA [NM_001165899]	*PDE4D *	0.289	**−0.15**
A_33_P3759611	Phosphodiesterase 4C, cAMP-specific (PDE4C), transcript variant 1, mRNA [NM_000923]	*PDE4C *	0.110	**−0.32**
A_23_P74278	Phosphodiesterase 4B, cAMP-specific (PDE4B), transcript variant d, mRNA [NM_001037341]	*PDE4B *	0.039	**−0.20**
A_24_P322474	Phosphodiesterase 4A, cAMP-specific (PDE4A), transcript variant 4, mRNA [NM_006202]	*PDE4A *	0.336	**−0.23**
A_23_P401106	Phosphodiesterase 2A, cGMP-stimulated (PDE2A), transcript variant 1, mRNA [NM_002599]	*PDE2A *	0.511	**−0.06**

**Table 2:** Alteration of genes related to the potassium channel.

Probe name	Description	Gene symbol	*P*-value	Fold change
A_33_P3338793	Potassium voltage-gated channel, S+C38haw-related subfamily, member 3 (KCNC3), mRNA [NM_004977]	*KCNC3 *	0.004	−**0.93 **
A_33_P3415012	Potassium voltage-gated channel, shaker-related subfamily, member 6 (KCNA6), mRNA [NM_002235]	*KCNA6 *	0.004	−**0.59 **
A_33_P3395823	Potassium voltage-gated channel, KQT-like subfamily, member 2 (KCNQ2), transcript variant 5, mRNA [NM_172109]	*KCNQ2 *	0.001	−**0.74 **
A_23_P154855	Potassium voltage-gated channel, Isk-related family, member 1 (KCNE1), transcript variant 2, mRNA [NM_000219]	*KCNE1 *	0.014	−**0.72 **
A_23_P119573	Potassium intermediate/small conductance calcium-activated channel, subfamily N, member 1 (KCNN1), mRNA [NM_002248]	*KCNN1 *	0.034	−**0.85 **
A_33_P3255131	Potassium channel tetramerisation domain containing 19 (KCTD19), mRNA [NM_001100915]	*KCTD19 *	0.011	−**0.91 **
